# One hour post-load plasma glucose and 3 year risk of worsening fasting and 2 hour glucose tolerance in the RISC cohort

**DOI:** 10.1007/s00125-018-4798-5

**Published:** 2018-12-29

**Authors:** Melania Manco, Andrea Mari, John Petrie, Geltrude Mingrone, Beverley Balkau, for the EGIR-RISC study group

**Affiliations:** 1 Research Area for Multifactorial Diseases and Complex Phenotypes, Bambino Gesù Children’s Hospital, Viale Ferdinando Baldelli 38, 00146 Rome, Italy; 2 CNR Institute of Neuroscience, Padua, Italy; 3 Institute of Cardiovascular and Medical Sciences, BHF Glasgow Cardiovascular Research Centre, University of Glasgow, 126 University Place, Glasgow, G12 8TA UK; 4 Department of Internal Medicine, IRCCS Policlinico Universitario A. Gemelli, Catholic University of Sacred Heart, Rome, Italy; 5 Department of Diabetes, King’s College London, London, UK; 6 CESP Centre for Research in Epidemiology and Population Health, Univ Paris-Saclay, Univ Paris Sud, Villejuif, France

**Keywords:** Diagnostic criteria, Post-challenge glucose, Post-load glucose, Prediabetes, Prediabetes phenotype, Progression

*To the Editor*: The 1 h post-load plasma glucose (1hPG) measurement has the potential to serve as a sensitive screening tool for identifying people who, despite having normal glucose tolerance (NGT), are at high-risk of developing type 2 diabetes over the next few years [1, 2]. Screening would be timely, as beta cells are still functional and lifestyle and drug interventions may be effective in delaying diabetes onset.

High 1hPG has been found to perform as well as the 2 h post-load plasma glucose (2hPG) measurement in predicting type 2 diabetes risk after median follow-up times of 9 and 13 years [3]. In a 33 year study, it was not only a better predictor of incident diabetes, but also of diabetes complications and mortality [4]. Robust evidence from the Botnia study and Malmö Prevention Project cohorts supports 1hPG as the best simple variable predicting incident type 2 diabetes, in comparison with other indices [1].

In a cross-sectional study, we previously described reduced euglycaemic clamp insulin sensitivity and impaired beta cell glucose sensitivity (BCGS) in people with NGT but with high 1hPG in 1205 healthy participants in the European Group for the Study of Insulin Resistance (EGIR) cohort: Relationship between Insulin Sensitivity and Cardiovascular Risk (RISC) [2]. There was a significant decreasing trend in insulin sensitivity from NGT with low 1hPG, to NGT with high 1hPG to impaired glucose tolerance (IGT: 2hPG: 7.8–11.1 mmol/l); BCGS was significantly higher in those with NGT and low 1hPG, in comparison with NGT and high 1hPG or IGT. This analysis of NGT included people without IGT and with a fasting plasma glucose (FPG) <6.1 mmol/l, the WHO definition of impaired fasting glucose (IFG) [2]. In our previous cross-sectional analysis of the baseline population, a 1hPG of 8.95 mmol/l was the ‘optimal’ cut-point (maximising [sensitivity + specificity]) associating 1hPG with prevalent IGT [2].

We now report 3 year longitudinal data from 797 participants with NGT at baseline, who had complete baseline and follow-up glucose data (see Table 1). Participants in the RISC study gave written informed consent. Ethics committee approval was obtained in each centre. The study was carried out in accordance with the Declaration of Helsinki as revised in 2008. The RISC Project Management Board approved the present analysis. In the present analysis, the definition of NGT was based on the ADA 2003 criteria (FPG <5.6 mmol/l and 2hPG <7.8 mmol/l, and not being treated for diabetes). The glucose tolerance status had worsened after 3 years for 183 people (23%): 40 had normal FPG but IGT; 117 had normal 2hPG but IFG (defined as FPG 5.6–6.9 mmol/l); 26 had both high FPG and 2hPG (including one case of diabetes diagnosed on the basis of FPG, and one on the basis of 2hPG). There was a higher percentage of progression to isolated IFG than to isolated IGT (15% vs 5%), with 3% showing progression on both FPG and 2hPG.

**Table 1:** Baseline characteristics and 3 year changes, according to baseline low or high 1hPG and 3 year progression to FPG ≥ 5.6 mmol/l and/or 2hPG ≥ 7.8 mmol/l

	Low 1hPG <8.6 mmol/l (*n* = 620)				High 1hPG ≥8.6 mmol/l (*n* = 177)			
	Non-progressor	Progressor	*p* values		Non-progressor	Progressor	*p* values	
	*n* = 505 (81%)	*n* = 115 (19%)	Univariate	Adjusted	*n* = 109 (62%)	*n* = 68 (38%)	Univariate	Adjusted
At baseline								
Men (%)	37	45	0.1133	0.1243	48	68	0.0101	0.0152
Age (years)	42 (36–49)	46 (39–54)	<0.0001	<0.0001	46 (38–51)	46 (40–54)	0.3933	0.2194
BMI (kg/m^2^)	23.8 (21.8–26.2)	25.2 (23.4–27.8)	0.0001	0.0008	24.7 (23.2–27.1)	26.1 (23.5–28.6)	0.0383	0.0813
Diabetes in family (%)	21	23	0.6402	0.6938	28	36	0.2360	0.3143
Smoker (%)	25	25	0.9938	0.4434	36	33	0.6851	0.8395
FPG (mmol/l)	4.9 (4.6–5.1)	5.2 (4.9–5.4)	<0.0001	<0.0001	5.1 (4.8–5.3)	5.2 (5.1–5.4)	0.0025	0.0154
2hPG (mmol/l)	5.0 (4.3–5.8)	5.7 (4.8–6.3)	<0.0001	<0.0001	6.1 (5.3–6.9)	6.4 (5.7–7.0)	0.1748	0.0724
LDL-cholesterol (mmol/l)	2.8 (2.2–3.3)	2.9 (2.5–3.4)	0.0576	0.8499	2.9 (2.4–3.3)	3.1 (2.6–3.6)	0.0496	0.1773
HDL-cholesterol (mmol/l)	1.5 (1.2–1.7)	1.3 (1.1–1.6)	0.0134	0.0563	1.4 (1.1–1.6)	1.3 (1.1–1.5)	0.1311	0.9096
Triacylglycerol (mmol/l)^a^	0.83 (0.62–1.14)	0.93 (0.68–1.21)	0.0941	0.9211	0.91 (0.70–1.15)	1.12 (0.78–1.66)	0.0066	0.1038
Systolic BP (mmHg)	115 (107–124)	119 (110–127)	0.0119	0.8486	119 (110–125)	122 (114–130)	0.0363	0.2529
Diastolic BP (mmHg)	73 (67–79)	75 (70–79)	0.0792	0.6799	75 (69–80)	77 (73–82)	0.0473	0.2378
Basal insulin secretion (pmol min^−1^ m^−2^)^a^	61 (49–78)	69 (54–92)	0.0029	0.0597	65 (48–91)	86 (68–100)	0.0005	0.0044
Total insulin secretion (nmol/m^2^)	36 (28–43)	39 (33–47)	0.0056	0.0623	44 (34–55)	46 (39–55)	0.1279	0.2629
BCGS (pmol min^−1^ m^−2^ mmol^−1^ l)^a^	133 (100–187)	134 (99–176)	0.7585	0.9362	90 (66–112)	86 (69–103)	0.9380	0.9380
Clamp insulin sensitivity (μmol min^−1^ kg_FFM_^−1^ nmol^−1^ l)^a^	143 (111–194)	134 (99–181)	0.1298	0.8733	133 (90–171)	95 (75–138)	0.0016	0.0481
OGIS (ml min^−1^ kg_FFM_^−1^)^a^	12.7 (11.1–14.5)	11.6 (10.2–12.9)	<0.0001	0.0001	10.5 (9.6–11.9)	9.9 (8.7–11.2)	0.0278	0.6382
Per cent change over 3 years								
BMI	1.0 (−2.1–4.9)	2.0 (−1.8–4.8)	0.7289	0.2461	1.5 (−2.3–3.7)	1.4 (0.1–5.1)	0.3518	0.3541
Basal insulin secretion^a^	−0.5 (−18–20)	14 (−5–38)	0.0092	0.0114	4.5 (−14–24)	11 (−5.8–32)	0.1049	0.0728
Total insulin secretion^a^	4.8 (−10–24)	15 (−3–39)	0.0050	0.0550	−5.3 (−22–12)	12 (−10–27))	0.1110	0.2288
BCGS^a^	−8.7 (−37–35)	−15 (−34–12)	0.3274	0.1556	24 (−3.7–74)	−2.4 (−21–48)	0.0302	0.0600
OGIS	−2.7 (−12–6.9)	−14 (−22–−4.5)	<0.0001	<0.0001	7.7 (−6.9–20)	−13 (−18–−3.5)	<0.0001	<0.0001

Data are median (quartile 1–quartile 3) or % for both baseline variables and per cent change over 3 years variables

Progressors were participants who presented with FPG ≥5.6 mmol/l, or 2hPG ≥7.8 mmol/l or both conditions after 3 years of follow-up

*p* values used logistic regression, unadjusted and adjusted for sex, age and BMI

^a^Logarithms, base *e*, were used in the logistic regression analyses

FFM, fat-free mass

In the population currently being studied over 3 years of follow-up, as described in Table 1, the ‘optimal’ cut-point associated with a worsening glucose status was 7.6 mmol/l, corresponding to 306 (38%) of our NGT population. After adjusting for sex, age and BMI, the corresponding cut-point was 6.2 mmol/l, corresponding to 526 (66%) of our population. These frequencies of people at risk of diabetes are probably too high for these cut-points to be used in practice. A petition has been published proposing a 1hPG of 8.6 mmol/l be used as a cut-point for diagnosing IGT, based on a number of large population based studies [5]; this cut-point identified 177 (22%) in our population.

In the present analysis, the percentage of people whose glucose status progressed according to 1hPG (electronic supplementary material [ESM] Fig. 1) showed a linear relation—the higher the 1hPG, the higher the percentage that progressed—but there is no clear threshold for defining a cut-point. However, comparing people with a 1hPG ≥8.6 mmol/l with those below this cut-point, the OR of progression was 2.74 (95% CI 1.90, 3.95); after adjusting the logistic regression for sex, age and BMI the OR was 2.19 (1.49, 3.20) and this remained statistically significant after adjusting for either FPG or 2hPG. The 1hPG associated with progressing according to either FPG or 2hPG, or both, had a C statistic of 0.67, and this was not significantly different from those of FPG (0.71) or 2hPG (0.65), using the DeLong test, in keeping with previous studies [2, 3].

In the current group of 797 participants we present, as medians (interquartile range) or %, the metabolic features of individuals with NGT whose glucose status progressed (‘progressors’) vs those who did not (‘non-progressors’) according to 1hPG (< and ≥8.6 mmol/l) at baseline (Table 1). Comparisons between progressors and non-progressors were made by logistic regression, unadjusted, and adjusted for sex, age and BMI. In progressors from both NGT groups, after adjusting for sex, age and BMI, a higher baseline FPG was the only common statistically significant risk factor; however in the low 1hPG group, progressors were older, the 2hPG was higher and the oral glucose insulin sensitivity (OGIS) index [6] was lower in progressors than non-progressors; for the high 1hPG group, basal insulin secretion was higher and the clamp measure of insulin sensitivity lower in progressors than in non-progressors, indicating the importance of these two factors. Over three years of follow-up, the OGIS index decreased more in progressors in both 1hPG groups, while in the low 1hPG group, basal insulin secretion increased more in progressors (Table 1). The sample sizes in our data are not large, particularly for the high 1hPG group; we can note that over three years the BCGS decreased more in progressors than in non-progressors in the high 1hPG group (*p =* 0.0600).

More people progressed in relation to an increase in FPG than in 2hPG, 143 (18%) vs 66 (8%) (with 26 of these progressing in relation to both), so the metabolic features described in the paragraph above for progressors reflect more the metabolic impairment of participants who developed isolated IFG rather than isolated IGT or combined IFG and IGT; indeed we observed a higher clamp insulin sensitivity for isolated IFG than for isolated IGT, in both 1hPG groups (ESM Tables 1 and 2). In the low 1hPG group, comparing people who progressed to isolated IFG or isolated IGT over three years, basal insulin secretion increased more, total insulin secretion less and OGIS decreased less in those who progressed to isolated IFG; with the small sample sizes in the high 1hPG group, no statistically significant differences were seen, but changes were in the same direction (ESM Tables 1 and 2).

These new data from the RISC study further support the notion that high 1hPG is associated with an increased risk of IGT, and also of IFG, and it represents an intermediate risk category between IFG and IGT, supporting the case to rehabilitate the 1hPG test for use in the prediction of type 2 diabetes risk.

The RISC study provides insight on mechanisms involved in the deterioration of glucose homeostasis in individuals at risk of type 2 diabetes. The balance between insulin secretion and insulin sensitivity is central along the progression pathway to overt diabetes in a continuum of risk. Data from other cohort studies with similar measures are now required to validate our results.

### Electronic supplementary material

ESM (PDF 231 kb)

## Data Availability

The RISC data base is open to other researchers for projects approved by the Project Management Board. Requests should be addressed to egir@med.unipi.it.

## Appendix

### EGIR-RISC Investigators

#### EGIR-RISC recruiting centres

**Amsterdam, the Netherlands:** RJ Heine, J Dekker, S de Rooij, G Nijpels, W Boorsma

**Athens, Greece**: A Mitrakou, S Tournis, K Kyriakopoulou, P Thomakos

**Belgrade, Serbia**: N Lalic, K Lalic, A Jotic, L Lukic, M Civcic

**Dublin, Ireland**: J Nolan, TP Yeow, M Murphy, C DeLong, G Neary, MP Colgan, M Hatunic

**Frankfurt, Germany:** T Konrad, H Böhles, S Fuellert, F Baer, H Zuchhold

**Geneva, Switzerland:** A Golay, E Harsch Bobbioni,V Barthassat, V Makoundou, TNO Lehmann, T Merminod

**Glasgow, Scotland, UK**: JR Petrie, C Perry, F Neary, C MacDougall, K Shields, L Malcolm

**Kuopio, Finland:** M Laakso, U Salmenniemi, A Aura, R Raisanen, U Ruotsalainen, T Sistonen, M Laitinen, H Saloranta

**London, England, UK:** SW Coppack, N McIntosh, J Ross, L Pettersson, P Khadobaksh

**Lyon, France**: M Laville, F Bonnet (now Rennes), A Brac de la Perriere, C Louche-Pelissier, C Maitrepierre, J Peyrat, S Beltran, A Serusclat

**Madrid, Spain:** R Gabriel, EM Sánchez, R Carraro, A Friera, B Novella

**Malmö, Sweden (1):** P Nilsson, M Persson, G Östling, **(2):** O Melander, P Burri

**Milan, Italy:** PM Piatti, LD Monti, E Setola, E Galluccio, F Minicucci, A Colleluori

**Newcastle-upon-Tyne, England, UK:** M Walker, IM Ibrahim, M Jayapaul, D Carman, C Ryan, K Short, Y McGrady, D Richardson

**Odense, Denmark:** H Beck-Nielsen, P Staehr, K Højlund, V Vestergaard, C Olsen, L Hansen

**Perugia, Italy:** GB Bolli, F Porcellati, C Fanelli, P Lucidi, F Calcinaro, A Saturni

**Pisa, Italy:** E Ferrannini, A Natali, E Muscelli, S Pinnola, M Kozakova, A Casolaro, BD Astiarraga

**Rome, Italy:** G Mingrone, C Guidone, A Favuzzi, P Di Rocco

**Vienna**, **Austria**: C Anderwald, M Bischof, M Promintzer, M Krebs, M Mandl, A Hofer, A Luger, W Waldhäusl, M Roden

**Project Management Board**: B Balkau (Villejuif, France), F Bonnet (Rennes, France), SW Coppack (London, England, UK), JM Dekker (Amsterdam, the Netherlands), E Ferrannini (Pisa, Italy), A Mari (Padova, Italy), A Natali (Pisa, Italy), J Petrie (Glasgow, Scotland, UK), M Walker (Newcastle, England, UK)

#### Core laboratories and reading centres

**Lipids:** Dublin, Ireland: P Gaffney, J Nolan, G Boran

**Hormones:** Odense, Denmark: C Olsen, L Hansen, H Beck-Nielsen

**Albumin:creatinine:** Amsterdam, the Netherlands: A Kok, J Dekker

**Genetics:** Newcastle-upon-Tyne, England, UK: S Patel, M Walker

**Stable isotope laboratory:** Pisa, Italy: A Gastaldelli, D Ciociaro

**Ultrasound reading centre:** Pisa, Italy: M Kozakova

**ECG reading:** Villejuif, France: MT Guillanneuf

**Actigraph:** Villejuif, France: B Balkau, L Mhamdi

**Data Management:** Villejuif, France, Padova, and Pisa, Italy: B Balkau, A Mari, L Mhamdi, L Landucci, S Hills, L Mota

**Mathematical modelling and website management:** Padova, Italy: A Mari, G Pacini, C Cavaggion, A Tura

**Coordinating office:** Pisa, Italy: SA Hills, L Landucci, L Mota

Further information on the EGIR-RISC study and participating centres can be found on www.egir.org.

## Abbreviations

BCGS: Beta cell glucose sensitivity
EGIR: European Group for the Study of Insulin Resistance
FPG: Fasting plasma glucose
IFG: Impaired fasting glucose
IGT: Impaired glucose tolerance
NGT: Normal glucose tolerance
OGIS: Oral glucose insulin sensitivity
1hPG: 1 h post-load plasma glucose
2hPG: 2 h post-load plasma glucose
RISC: Relationship between Insulin Sensitivity and Cardiovascular Risk
