# Supplementary material for: One hour post-load plasma glucose and 3 year risk of worsening fasting and 2 hour glucose tolerance in the RISC cohort
Source: Diabetologia. 2018 Dec 29;62(3):544–8. doi: 10.1007/s00125-018-4798-5 (PMC6428784; doi:10.1007/s00125-018-4798-5)

Electronic Supplementary Material (ESM)

One hour post-load plasma glucose and three-year risk of worsening fasting and 2 hour glucose tolerance in the RISC cohort

Melania Manco[1], Andrea Mari[2], John Petrie[3], Geltrude Mingrone[4,5], Beverley Balkau[6], for the EGIR-RISC study group

[1] Research Area for Multifactorial Diseases and Complex Phenotypes, Bambino Gesù Children's Hospital, Rome. Italy.

[2] CNR Institute of Neuroscience, Padua, Italy.

[3] Institute of Cardiovascular and Medical Sciences, University of Glasgow. United Kingdom.

[4] Department of Internal Medicine, IRCCS Policlinico Universitario A. Gemelli, Catholic University of Sacred Heart, ~~Largo Agostino Gemelli 8, 00168~~, Rome, Italy and

[5] Department of Diabetes, King's College London, United Kingdom.

[6] CESP Centre for Research in Epidemiology and Population Health, Univ Paris-Saclay, Univ Paris Sud, ~~UVSQ, UMRS 1018,~~ Villejuif, France.

ESM Table 1 Anthropometrics and metabolic characteristics of RISC participants who had baseline 1hPG <8.6 mmol/l and progressed over 3 years to isolated IFG, isolated IGT, both.

Low 1hPG < 8.6 mmol/l

	Non progressor	Progressor Isolated high FPG	*P-values* comparing isolated high FPG and isolated high 2hPG progressor		Progressor isolated 2hPH	Progressor both FPG & 2hPG high	*P-values* comparing four groups	
	n=505 (81%)	n=81 (13%)	univariate	adjusted†	n=26 (4%)	n=8 (1%)	univariate	adjusted†
**At baseline**								
Men	37%	49%			35%	38%	0.2138	2496
Age (years)	42 (36;49)	46 (38;53)	0.1844	0.9976	46 (41;55)	44 (39;54)	0.0003	0.0006
BMI (kg/m²)	23.8 (21.8;26.2)	25.2 (23.4;27.6)	0.2541	0.2840	24.7 (23.2;27.7)	28.2 (24.6;29.8)	0.0006	0.0034
Diabetes in family	21%	22%			19%	50%	0.3204	0.3456
Smoker	25%	28%			19%	12%	0.6677	0.5135
Fasting plasma glucose (mmol/l)	4.9 (4.6;5.1)	5.3 (5.0;5.4)	0.0061	0.0101	5.0 (4.7;5.2)	5.0 (4.9;5.3)	<.0001	<.0001
2 hour plasma glucose (mmol/l)	5.0 (4.3;5.8)	5.3 (4.6;6.0)	0.0050	0.0068	6.0 (5.7;6.8)	6.5 (6.0;6.9)	<.0001	<.0001
LDL-cholesterol (mmol/l)	2.8 (2.2;3.3)	2.8 (2.2;3.3)			3.1 (2.8;3.7)	3.0 (2.6;3.0)	0.0630	0.1429
HDL-cholesterol (mmol/l)	1.5 (1.2;1.7)	1.3 (1.1;1.7)			1.3 (1.1;1.6)	1.3 (1.1;1.6)	0.0796	0.0871
Triglycerides (mmol/l) [a]	0.83 (0.62;1.14)	0.93 (0.68;1.14)			1.00 (0.65;1.32)	0.81 (0.63;1.41)	0.3323	0.6122
Systolic blood pressure (mmHg)	115 (107;124)	120 (111;128)			116 (109;126)	120 (112;128)	0.0879	0.9839
Diastolic blood pressure (mmHg)	73 (67;79)	75 (70;79)			74 (70;77)	77 (70;84)	0.2285	0.8246
Basal insulin secretion ($pmol·min^{-1}·m^{-2}$)*	61 (49;78)	68 (53;92)			71 (58;92)	70 (68;96)	0.0134	0.1289
Total insulin secretion	36	37	0.0069	0.0057	45	40	0.0015	0.0075

(nmol·m^{-2})	(28;43)	(33;44)			(37;57)	(32;43))		
Beta cell glucose sensitivity (pmol·min^{-1}·m^{-2}·mmol/l) [a]	133 (100;187)	136 (108;188)			119 (97;153)	97 (90;123)	0..4238	0.3212
Clamp insulin sensitivity (μmol·min·kg$_{FFM}$·nmol·l) [a]	143 (111;194)	145 (110;197)	0.0003	<.0001	105 (81;123)	108 (92;150)	0.0002	<.0001
OGIS (ml·min^{-1}·m^{2})*	12.7 (11.1;14.5)	11.8 (10.7;12.9)	0.1261	0.1973	11 0 (9.6;13.0)	11.0 (9.9;12.4)	<.0001	<.0001

**Percent change over 3 years**								
BMI	1.0 (-2.1;4.9)	1.3 (-1.8;4.3)			2.2 (-0.5;5.4)	5.0 (-0.6;8)	0.4144	0.2552
Basal insulin secretion [a]	-0.5 (-18;20)	19.0 (-1;46)	0.0075	0.0139	-6.5 (-25;25)	10.3 (1;17)	0.0001	0.0001
Total insulin secretion [a]	4.8 (-10;24)	12.7 (-3.8;39)	0.0089	0.0074	16.6 (3.3;40)	22.8 (1.5;49)	0.0017	0.0088
Beta cell glucose sensitivity [a]	-8.7 (-37;35)	-8.7 (-30; 30)			-33 (-41;-4.1)	-22 (-31;0.4)	0.1771	0.1233
OGIS	-2.7 (-12;7)	-9.7 (-21;-2.4)	0.0097	0.0082	-17.2 (-28;-11)	-20.8 (-25; -12)	<.0001	<.0001

Data are reported as median values (quartile1; quartile 3) or % of baseline characteristics and of per cent change over 3 years.

p values used multinomial regression, unadjusted and †adjusted for sex, age and BMI. The isolated high FPG and isolated high 2hPG are compared statistically in the case where the comparison over four groups was statistically significant.

[a] Logarithms, base, were used in the logistic regression analyses

FFM, fat-free mass

Progressors on isolated IFG were participants who presented with FPG ≥ 5.6 mmol/l, but 2hPG <7.8 mmol/l;
Progressors on isolated IGT were participants who presented with FPG < 5.6 mmol/l, but 2hPG ≥ 7.8 mmol/l;
Progressors on both FPG and 2hPG high, had FPG ≥ 5.6 mmol/l and 2hPG ≥7.8 mmol/l.

ESM Table 2 Anthropometrics and metabolic characteristics of RISC participants who had baseline 1hPG ≥8.6 mmol/l and progressed over 3 years to isolated IFG, isolated IGT, both.

	Non-progressor	Progressor isolated high FPG	*P-values* *comparing isolated high FPG and isolated high 2hPG progressor*		Progressor isolated 2hPH	Progressor both FPG & 2hPG high	*P-values* *comparing four groups*	
	n=109 (62%)	n=36 (20%)	univariate	adjusted†	n=14 (8%)	n=18 (10%)	univariate	adjusted†
**At baseline**								
Men	48%	78%	0.0608	0.0798	50%	61%	0.0223	0.0204
Age (years)	46 (38;51)	46 (43;54)			42 (38;50)	48 (39;55)	0.4735	0.2613
BMI (kg/m²)	24.7 (23.2;27.1)	25.8 (23.6;28.0)	0.6579	0.9244	25.2 (21.8;28.1)	28.4 (24.5;31.2)	0.0332	0.0484
Diabetes in family	28%	47%			29%	22%	0.1625	0.1001
Smoker	36%	17%			43%	56%	0.0443	0.0830
Fasting plasma glucose (mmol/l)	5.1 (4.8;5.3)	5.3 (5.1;5.4)			5.2 (4.9;5.3)	5.3 (5.1;5.3)	0.0265	0.1171
2 hour plasma glucose (mmol/l)	6.1 (5.3;6.9)	5.9 (5.3;6.9)	0.0210	0.0504	6.9 (6.1;7.4)	6.7 (5.8;7.0)	0.0357	0.0465
LDL-cholesterol (mmol/l)	2.9 (2.4;3.3)	3.2 (2.5;3.7)			3.1 (2.6;4.0)	2.9 (2.8;3.5)	0.1950	0.2096
HDL-cholesterol (mmol/l)	1.4 (1.1;1.6)	1.3 (1.1;1.5)			1.3 (1.2;1.9)	1.2 (1.0;1.5)	0.2594	0.5388
Triglycerides (mmol/l) [a]	0.91 (0.70;1.15)	1.06 (0.69;1.95)			0.99 (0.87;1.16)	1.29 (0.89;2.27)	0.0144	0.1687
Systolic blood pressure (mmHg)	119 (110;125)	125 (117;130)			118 (112;119)	122 (116;130)	0.0114	0.1120
Diastolic blood pressure (mmHg)	75 (69;80)	78 (74;84)			75 (72;77)	78 (71;82)	0.0712	0.3403
Basal insulin secretion ($pmol\cdot min^{-1}\cdot m^{-2}$)*	65 (48;91)	76 (66;95)	0.6597	0.2730	82 (72;94)	96 (82;128)	0.0019	0.0247
Total insulin secretion ($nmol\cdot m^{-2}$)	44 (34;55)	45 (38;52)			47 (43;53)	49 (42;58)	0.3275	0.6093

Beta cell glucose sensitivity (pmol·min^{-1}·m^{-2}·mmol/l) [a]	90 (66;112)	83 (72;97)			89 (82;103)	86 (56;111)	0.8684	0.8604
Clamp insulin sensitivity (µmol·min·kg$_{FFM}$·nmol·l) [a]	133 (90;171)	100 (82;134)	0.4695	0.0212	79 (60;153)	89 (80;157)	0.0151	0.0488
OGIS (ml·min^{-1}·m^{2})	10.5 (9.6;11.9)	9.9 (8.8;11.2)			9.4 (8.7;12.0)	10.1 (9.1;11.1)	0.1785	0.6469
**Percent change over 3 years**								
BMI	1.5 (-2.3;3.7)	1.1 (-2.6;2.8)	0.1416	0.1945	1.8 (0.4;6.1)	4.6 (2.2;6.6)	0.0596	0.0353
Basal insulin secretion [a]	4.5 (-14;24)	12 (-5.8;34)			-2.3 (-17;26)	18.0 (-4.7;42)	0.2330	0.1780
Total insulin secretion [a]	-5.3 (-22;12)	-0.2 (-18;20)			28 (22;32)	16 (-1.4;34)	0.2818	0.5469
Beta cell glucose sensitivity [a]	24 (-3.7;74)	-9.9 (-20;50)			9.6 (-12;30)	-1.9 (-38;53)	0.1328	0.2147
OGIS	7.7 (-6.9;20)	-5.9 (-16, 4.1)	0.0835	0.0902	-14 (-17;-9.3)	-19 (-23;-13)	<.0001	<.0001

Data are reported as median values (quartile1; quartile 3) or % of baseline characteristics and of per cent change over 3 years.

p values used multinomial regression, unadjusted and †adjusted for sex, age and BMI. The isolated high FPG and isolated high 2hPG are compared statistically in the case where the comparison over four groups was statistically significant.

[a] Logarithms, base, were used in the logistic regression analyses

FFM, fat-free mass

Progressors on isolated IFG were participants who presented with FPG ≥ 5.6 mmol/l, but 2hPG <7.8 mmol/l;

Progressors on isolated IGT were participants who presented with FPG < 5.6 mmol/l, but 2hPG ≥ 7.8 mmol/l;

Progressors on both FPG and 2hPG high, had FPG ≥ 5.6 mmol/l and 2hPG ≥7.8 mmol/l.

Supplementary Figure

Percentage of people progressing to incident glucose intolerance (fasting plasma glucose ≥5.6 mmol/l and/or 2 hour plasma glucose ≥7.8 mmol/l) over a three year follow-up of 797 people from the EGIR-RISC Study. The thresholds of 1hPG potentially associated with risk of progression to glucose intolerance were 6.2; 7.5; 8.6; 8.95 mmol/l)

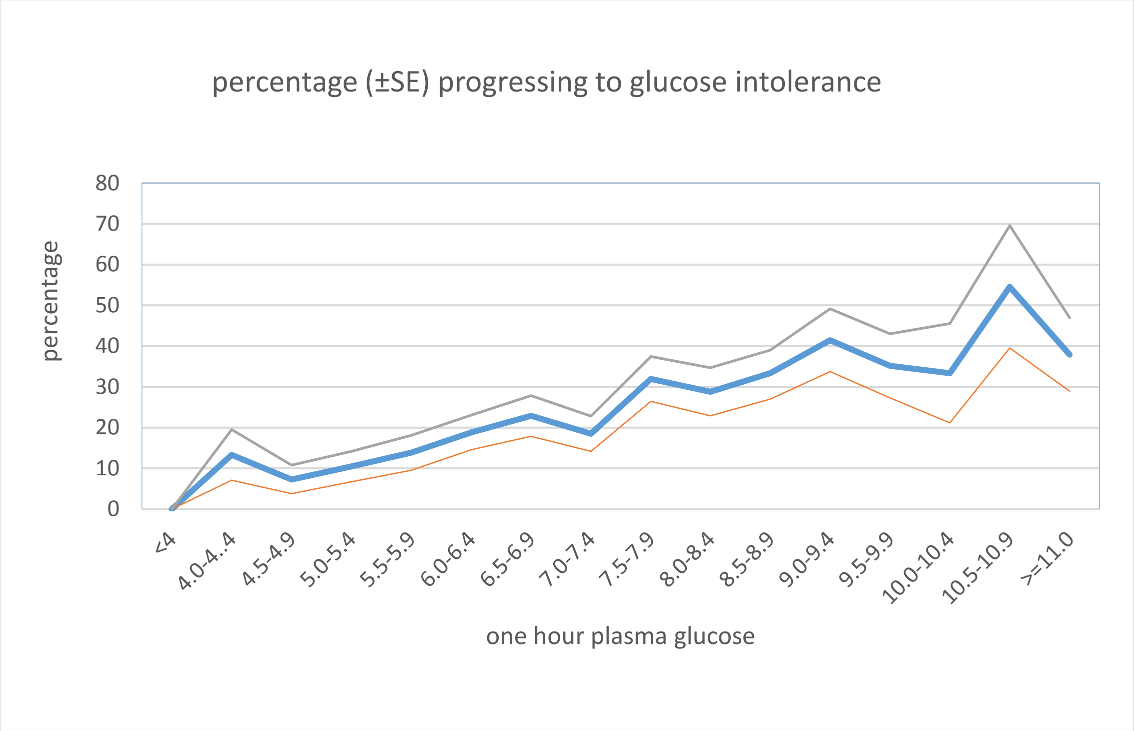

Supplement: Supplementary file 1 — (PDF 231 kb) [file 125_2018_4798_MOESM1_ESM.pdf]
